# Strategies for facilitating the delivery of cluster randomized trials in hospitals: A study informed by the CFIR-ERIC matching tool

**DOI:** 10.1177/17407745211001504

**Published:** 2021-04-16

**Authors:** Arielle Weir, Justin Presseau, Simon Kitto, Ian Colman, Simon Hatcher

**Affiliations:** 1 School of Epidemiology and Public Health, University of Ottawa, Ottawa, ON, Canada; 2 Clinical Epidemiology Program, Ottawa Hospital Research Institute, Ottawa, ON, Canada; 3 Department of Innovation in Medical Education, Faculty of Medicine, University of Ottawa, Ottawa, ON, Canada

**Keywords:** Cluster randomized controlled trial, Consolidated Framework for Implementation Research, hospitals, implementation, CFIR-ERIC matching, Expert Recommendations for Implementing Change

## Abstract

**Background:**

Recruitment and engagement of clusters in a cluster randomized controlled trial can sometimes prove challenging. Identification of successful or unsuccessful strategies may be beneficial in guiding future researchers in conducting their cluster randomized controlled trial. This study aimed to identify strategies that could be used to facilitate the delivery of cluster randomized controlled trials in hospitals.

**Methods:**

The study employed the Consolidated Framework for Implementation Research–Expert Recommendations for Implementing Change matching tool. The barriers and enablers to cluster randomized controlled trial conduct identified in our previously conducted studies served as a means of determinant identification for the conduct of cluster randomized controlled trials. These determinants were mapped to Consolidated Framework for Implementation Research constructs and then matched to Expert Recommendations for Implementing Change compilation strategies using the Consolidated Framework for Implementation Research–Expert Recommendations for Implementing Change matching tool.

**Results:**

The Expert Recommendations for Implementing Change strategies matched to at least one determinant Consolidated Framework for Implementation Research construct were as follows: (1) ‘Identify and prepare champions’, (2) ‘Conduct local needs assessment’, (3) ‘Conduct educational meetings’, (4) ‘Inform local opinion leaders’, (5) ‘Build a coalition’, (6) ‘Promote adaptability’, (7) ‘Develop a formal implementation blueprint’, (8) ‘Involve patients/consumers and family members’, (9) ‘Obtain and use patients/consumers and family feedback’, (10) ‘Develop educational materials’, (11) ‘Promote network weaving’, (12) ‘Distribute educational materials’, (13) ‘Access new funding’ and (14) ‘Develop academic partnerships’.

**Conclusion:**

This study was intended as a step in the research agenda aimed at facilitating cluster randomized controlled trial delivery in hospitals and can act as a resource for future researchers when planning their cluster randomized controlled trial, with the expectation that the strategies identified here will be tailored to each context.

## Introduction

Cluster randomized trials offer advantages over individually randomized trials;^1^ however, they also present some challenges to the organization and conduct of the trials.^2^ Challenges in the delivery of trials can arise before patient recruitment and/or intervention launch.^3,4^ Recruitment and continued engagement of the clusters as a whole may prove difficult, with poor response and participation from the intended sites often encountered by researchers.^5^ In part due to the randomization of entire clusters rather than individuals, the loss of individual sites and/or an inability to recruit the required sample size (whether by a site refusing to participate in the study or dropping out of the study for any reason after randomization) can prove more detrimental to adequately power the trial than the loss of power that would be experienced when losing a site in an individually randomized trial.^6^ There is usually a smaller number of sites in a cluster randomized trials than there would be individuals in an individually randomized controlled trial. The inability to recruit an adequate number of sites or the loss of a site post-randomization can pose threats to the statistical analysis, to the conduct of the trial and to the interpretation of the trial results.^2,6^

To guide researchers’ planning trials, recent literature has emphasized the need to consider more empirical and generalizable research into the conduct of trials themselves.^7^ The conduct of cluster randomized trials is often relegated to the internal expertise of a trial team rather than being informed by results reported as part of trials, making these processes effectively a ‘black box’.^3^ While the identification of barriers to implementation and development of strategies to facilitate the delivery of trials is prioritized in implementation science research,^8^–^11^ there remains a lack of knowledge linking theory-informed barriers and enablers to implementation strategies best suited to address them. To select and operationalize intervention strategies, methods of intervention strategy mapping can be used through a combination of theory and evidence from the literature.^12^

Barriers to conducting cluster randomized controlled trials may have commonalities between studies; however, there is currently limited literature or guidelines to assist research teams in identifying and circumventing these challenges. Therefore, the objective of this study was to identify strategies that could be used to facilitate the delivery of cluster randomized trials in hospitals.

## Methods

This study was guided by the Consolidated Framework for Implementation Research (CFIR)–Expert Recommendations for Implementing Change (ERIC) matching tool^13^ and our previously conducted scoping review^14^ and qualitative case study. The scoping review^14^ aimed to identify and describe the current literature surrounding the conduct of cluster randomized controlled trials in hospitals and to chart it to the domains of the CFIR (framework described below). The qualitative case study aimed to explore, from the perspective of the coordinating site of an ongoing cluster randomized trial in hospitals, factors influencing the launch and conduct of the trial, and again to chart the data to the domains of the CFIR.

### CFIR-ERIC matching tool

CFIR is a theoretical framework designed to assess potential contextual determinants of implementation^13^ and organizes commonalities and overlapping themes from various theories and concepts of implementation science.^13,15^ Using previously published research to provide a guide for effective implementation, the framework organizes 39 constructs into five domains (see Appendix A in the supplemental material for detailed definitions and constructs within the domains): (1) Intervention Characteristics – design features of the trial, (2) Outer Settings – external political and network influences, (3) Inner Settings – inner political and organizational influences within the sites, (4) Process – plans and procedures for implementation and (5) Characteristics of Individuals – personal factors and experiences of the people involved in implementation and conduct. The CFIR may be used as a framework to guide the development and evaluation of an implementation strategy, as well as for the investigation of barriers and enablers to trial implementation.^15^ Barriers and enablers to implementation may be evaluated within each of the five domains.^16^ CFIR does not provide explicit implementation strategies for operationalized use.^13,17^

The ERIC compilation is a list of 73 discrete implementation strategies that can be used as building blocks to plan the implementation and potentially address anticipated barriers^13,18^ (see Appendix B in the supplemental material for the strategies and their definitions). The development of the list involved several rounds of a modified-Delphi process with a panel of implementation science and clinical practice experts to generate consensus on strategies and their definitions.

To allow for the capacity to build an implementation plan using the ERIC compilation strategies matched to specific determinants from CFIR, the CFIR-ERIC matching tool was developed.^13,17^ This tool was developed by asking implementation science experts to rank the top seven ERIC strategies that, in their view, would address barriers categorized by each of the CFIR constructs.^13^ Strategies that were endorsed by ≥50% of the experts were deemed as ‘Level 1’ strategies, and strategies that were endorsed by 20% to 49.9% of the experts were deemed as ‘Level 2’ strategies. Based on the results of this study, the CFIR-ERIC matching tool was created and made available as an Excel download online at www.cfirguide.org.^16^ This tool allows users to select whether the determinant was deemed as relevant to the implementation strategy under question (yes/no) and generates an output table with the CFIR construct matched to the ERIC strategies with a percentage. This percentage indicates what percent of the experts queried in the tool development endorsed the strategy for the construct.

### Identification of determinants of cluster randomized trial delivery

Determinants of delivery of cluster randomized trials in hospitals were first identified through an assessment of the barriers and enablers to delivery and conduct of cluster randomized trials in hospitals from our previous scoping review^14^ and qualitative case study. The scoping review identified 22 articles, from which 18 CFIR constructs spanning four of the five CFIR domains were identified: Intervention Characteristics, Outer Settings, Inner Settings and Process.^14^ The qualitative case study included six interviewees, from which 24 constructs spanning all five CFIR domains were coded. Data from both the scoping review and the qualitative case study were coded by two independent reviewers. The ‘determinants’ of cluster randomized trial delivery in hospitals were identified from the themes that emerged in the scoping review and qualitative case study and were classified by whether they presented barriers or enablers to the delivery of the trials.

The CFIR-ERIC matching tool was used to generate an output that matched the CFIR constructs that had been identified as determinants of delivery of cluster randomized controlled trials in hospitals to ERIC compilation strategies. This matching was performed by one author (A.W.) with all the identified determinants, regardless of whether they were deemed as barriers or enablers for two reasons: (1) the determinants classified as enablers were generally framed as methods to overcome the perceived barriers and (2) implementation plans should ideally provide strategies that would support enablers. This article focused on Level 1 strategies, which are more likely to be effective in addressing the corresponding CFIR domains (based on expert consensus) compared to strategies that were not endorsed as Level 1 strategies.

## Results

**Figure 1.**
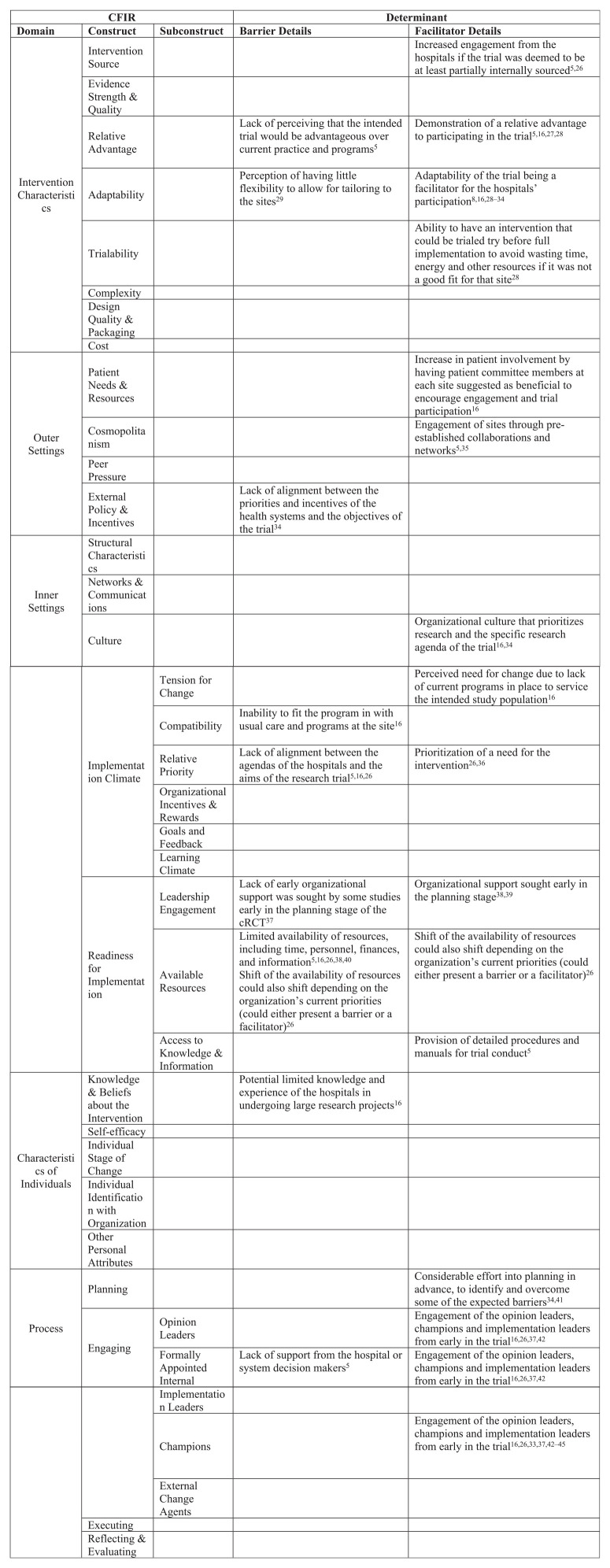
Determinants of cluster randomized controlled trial delivery identified from the scoping review and qualitative case study.

The determinants identified from the CFIR coded results of the scoping review and the qualitative case study are listed in Figure 1, along with their classification as barriers or enablers to delivering the trial.

In total, 19 constructs from all five of the CFIR domains were identified. Nine constructs, spanning all five of the CFIR domains, were categorized as barriers to delivery, and 16 spanning four of the CFIR domains were identified as enablers. Many of the constructs were categorized as both a barrier and an enabler to trial delivery.

**Figure 2.**
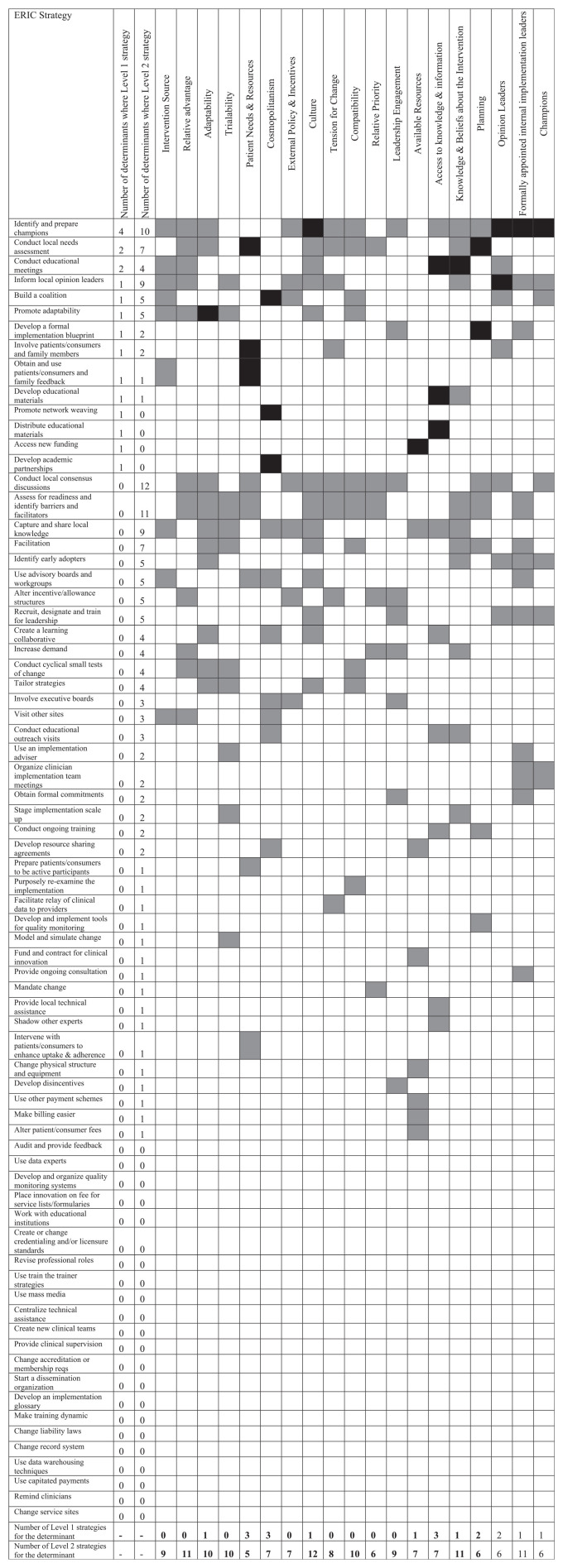
ERIC-CFIR matching of strategies to the determinants. Level 1 strategies (strategies endorsed by ≥50% of the experts) for each determinant are displayed in black. Level 2 strategies (endorsed by 20% to 49.9% of the experts) for each determinant are displayed in grey. Only ERIC strategies matched to relevant CFIR domains are displayed.

The mapping of ERIC strategies to identified CFIR domains is displayed in Figure 2. Strategies were ordered by the number of determinants for which the strategy was a Level 1 strategy, then by the number of determinants for which the strategy was a Level 2 strategy. The ERIC strategies (as ranked by the ordering described above) that were endorsed as a Level 1 strategy for at least one identified CFIR construct were as follows: (1) ‘Identify and prepare champions’, (2) ‘Conduct local needs assessment’, (3) ‘Conduct educational meetings’, (4) ‘Inform local opinion leaders’, (5) ‘Build a coalition’, (6) ‘Promote adaptability’, (7) ‘Develop a formal implementation blueprint’, (8) ‘Involve patients/consumers and family members’, (9) ‘Obtain and use patients/consumers and family feedback’, (10) ‘Develop educational materials’, (11) ‘Promote network weaving’, (12) ‘Distribute educational materials’, (13) ‘Access new funding’ and (14) ‘Develop academic partnerships’. The maximum number of determinants for which a strategy was listed as a Level 1 strategy was four (Identify and prepare champions). The CFIR constructs with the largest number of Level 1 endorsed strategies were Patient Needs & Resources, Cosmopolitan, and Access to Knowledge & Information, each with three Level 1 endorsed strategies.

### Case study example for the application of the identified ERIC strategies

To provide a practical application of these ERIC strategies, examples are linked to an ongoing cluster randomized controlled trial, the BEACON trial, which aims to investigate a clinical intervention involving a smartphone-assisted face-to-face therapy in men presenting to the emergency department for self-harm.^19^ Examples of the ERIC strategies identified in this article are presented either as an example that was performed in the trial or as a potential suggestion as lessons learned for the future:

1. ‘Identify and prepare champions’– Key personnel at the sites who were enthusiastic and engaged in the trial were identified and served as one point of contact for the trial. For future trials, there is the suggestion that patient champions may also be involved to drive the trial at the local level.
2. ‘Conduct local needs assessment’– In the BEACON trial, sites were not approached to determine whether they had a perceived clinical gap in the area of men who present to the emergency department with self-harm prior to the site being randomized. Approaching the sites in advance may have been beneficial in identifying whether there was a need for this intervention.
3. ‘Conduct educational meetings’– When a site was nearing the launch of the trial, the principal investigator and the research coordinators visited the sites in person with prepared educational materials to train the local site staff and to answer questions.
4. ‘Inform local opinion leaders’– Similar to Strategy 1 above, gatekeepers and local site members were identified and kept up to date with the stages of the trial.
5. ‘Build a coalition’– Partners and key leaders, including members from the sites, were identified early and participated in meetings as the development, launch and conduct of the trial ensued.
6. ‘Promote adaptability’– The intervention was allowed to be adapted to the local settings of a hospital with a high proportion of indigenous patients. The intervention was tailored to include indigenous images and audio. This site would not have agreed to participate had these adaptations not been possible.
7. ‘Develop a formal implementation blueprint’– Plans for the implementation of the intervention were outlined before the trial was started, which allowed for the sites to have an understanding as to whether the plans were appropriate for their site or whether adaptations would be required.
8. ‘Involve patients/consumers and family members’– Patients with lived experience were included in the trial design and were part of the coordinating team for BEACON as co-investigators.
9. ‘Obtain and use patients/consumers and family feedback’– Patients with lived experience provided input on the proposed trial, as well as tested the application before the trial was started to adapt it to the patients’ needs and views.
10. ‘Develop educational materials’– Material was developed throughout the trial to support ongoing educational needs, including the development of training videos for the smartphone application.
11. ‘Promote network weaving’– The principal investigator approached and engaged the head of psychiatry at one of the intended BEACON sites at a meeting from an association in which they were both members. This person then agreed to become a co-principal investigator on the study and was engaged throughout the trial conceptualization. This also demonstrated the utility of the early engagement of members from the sites to allow them to participate in designing the trial.
12. ‘Distribute educational materials’– Similar to Strategy 11 listed above, the material was distributed to the sites throughout the trial and during the site initiation visits.
13. ‘Access new funding’– Funding was secured through several grant applications, as well as specific funding from patient-oriented research organizations to support the inclusion of patients with lived experiences as co-investigators.
14. ‘Develop academic partnerships’– For future studies, the engagement of members and leaders of the sites may have benefitted from the clear development of academic partnerships, for example, in the form of co-authors on the resulting papers.

## Discussion

### Main findings

To the best of our knowledge, this is the first study using the CFIR-ERIC matching tool to identify potential strategies that may help to facilitate the delivery of cluster randomized trials in hospitals by addressing known barriers and enablers. The matching identified many ERIC compilation strategies that were listed for addressing CFIR coded determinants of cluster randomized trial delivery in hospitals.

Some of the identified strategies emphasize the potential value of clear planning for cluster randomized trials. The strategies ‘Conduct local needs assessment’ and ‘Develop a formal implementation blueprint’, while different in their overall focus, are both potentially crucial strategies to be employed while planning the cluster randomized trial.

Several of the identified strategies were similar in terms of their general focus. For example, three identified strategies were educational: ‘Conduct educational meetings’, ‘Develop educational materials’ and ‘Distribute educational materials’. Researchers planning for delivery of a cluster randomized controlled trials may be able to address all three strategies simultaneously. Guidelines for conducting educational meetings concluded that these strategies may have a modest effect on changing clinical behaviour, but only when they are planned, implemented and evaluated properly.^20^

In addition, several strategies involved engagement of people related to the intended sites: ‘Identify and prepare champions’, ‘Inform local opinion leaders’, ‘Involve patients/consumers and family members’ and ‘Obtain and use patients/consumers and family feedback’. The former two strategies involve engagement of relevant stakeholders early in planning for recruiting sites into cluster randomized trials, which has been emphasized as important.^21^–^26^ Meanwhile, the latter two strategies may emphasize the importance of engaging the patients/consumers to facilitate cluster randomized trial delivery, as they may be able to offer unique insights when planning and delivering the trial.

Although the effects of the identified ERIC compilation strategies on cluster randomized trial delivery and conduct were not evaluated in this matching study, systematic reviews from the Effective Practice and Organisation of Care (EPOC) group^27^ of the Cochrane Review Group have investigated the effects of various strategies for implementation of clinical trials and behaviour change in healthcare. While none of these reviews directly explored the effect of the strategies on cluster randomized trial delivery and conduct, they may offer some insight into whether some of these strategies can be effectively used in this context. These are further discussed below.

### Identified strategies in relation to EPOC group^27^ reviews

A strategy identified in this mapping study was ‘Inform local opinion leaders’. A review by the Cochrane EPOC group concluded that the strategy of recruiting local opinion leaders, when used alone or in combination with other strategies, can be effective to promote practice change.^28^ In this review, interventions that involved leveraging local opinion leaders showed minor absolute improvement in healthcare professionals’ compliance with evidence-based practice.

Three ERIC strategies surrounding educational aspects were identified to address some of the CFIR constructs deemed as determinants of cluster randomized controlled trial delivery in hospitals. A review by the EPOC group investigating the effects of educational outreach visits (defined as ‘a personal visit by a trained person to healthcare professionals in their own settings’^29^) on professional practice and healthcare outcomes concluded that this strategy has effects when used alone or in combination with other strategies on prescribing patterns.^29^ Another review, which explored the effect of printed educational materials on professional practice and healthcare outcomes, concluded that these strategies probably improve the practice by healthcare professionals compared to no intervention.^30^

While many ERIC strategies are aimed at addressing financial aspects of implementation, only one of these strategies was identified as relevant in this matching study: ‘Access new funding’. An ‘overview of reviews’ by the EPOC group investigating the effect of financial incentives in changing healthcare professional behaviours and patient outcomes demonstrated mixed effects of the strategy.^31^

### Future research

This study aimed to be a step in a research agenda aimed at facilitating cluster randomized trial delivery in hospitals. Researchers could use the list of strategies identified here as guidance to complete empirical evaluation of the effect of the strategies. These strategies were generated using the CFIR-ERIC matching tool and were not empirically tested. Therefore, their impact on the delivery of the cluster randomized trials should be empirically evaluated in conjunction with relevant stakeholders (e.g. trialists, trial funders, hospitals). In addition, researchers intending to use this list to develop implementation plans for their cluster randomized trial should aim to tailor it to their specific context and perform a needs assessment for their unique setting. Furthermore, it may be reasonable to investigate extending these strategies for recruitment of sites in non-hospital settings or in multicenter individually randomized trials.

### Limitations

The CFIR-ERIC matching tool is a recently produced guide, and the use of the tool in producing truly effective strategies has yet to be thoroughly evaluated. Potential limitations must be noted from the methods used to identify the determinants, which involved using results from the scoping review and the qualitative study. A major conclusion of the scoping review was the identification of a reporting gap surrounding the conduct and delivery of cluster randomized controlled trials. These aspects were rarely the focus of the published trials and were only described briefly. Therefore, some determinants may have been missed due to a lack of reporting. Furthermore, the qualitative case study was of one specific cluster randomized trial, and the determinants for that specific trial may not be empirically generalizable to other cluster randomized controlled trials in hospitals. However, this may have been mitigated by there being a large overlap in the determinants identified from the case study also being identified in the scoping review.

## Conclusion

This study aimed to identify strategies to facilitate the delivery of cluster randomized trials in hospitals using methods from implementation mapping. This study was informed by two studies previously conducted by our research group.^16^ A scoping review investigating the conduct of cluster randomized trials in hospitals and a qualitative case study exploring the coordinating site members’ perceptions of what was influencing the delivery of the group’s cluster randomized controlled trials in Ontario hospitals served as a needs assessment to inform this study. Barriers and enablers to cluster randomized trial delivery were coded to CFIR constructs, and the constructs were listed as determinants of cluster randomized trial delivery. These determinants were then mapped to strategies using the CFIR-ERIC matching tool.

This study was intended as a step in a needs assessment/gap analysis of the delivery of cluster randomized trials in hospitals and can act as a resource for future researchers when planning their cluster randomized trials, with the expectation that the strategies identified here will be tailored to each context. Future studies should attempt to further explore and evaluate the outcomes of the implementation plans derived from the strategies identified in this study.

## Supplemental Material

sj-pdf-1-ctj-10.1177_17407745211001504 – Supplemental material for Strategies for facilitating the delivery of cluster randomized trials in hospitals: A study informed by the CFIR-ERIC matching tool Click here for additional data file. Supplemental material, sj-pdf-1-ctj-10.1177_17407745211001504 for Strategies for facilitating the delivery of cluster randomized trials in hospitals: A study informed by the CFIR-ERIC matching tool by Arielle Weir, Justin Presseau, Simon Kitto, Ian Colman and Simon Hatcher in Clinical Trials
