# Supplementary material for: Strategies for facilitating the delivery of cluster randomized trials in hospitals: A study informed by the CFIR-ERIC matching tool
Source: Clin Trials. 2021 Apr 16;18(4):398–407. doi: 10.1177/17407745211001504 (PMC8290989; doi:10.1177/17407745211001504)
Supplement: sj-pdf-1-ctj-10.1177_17407745211001504 – Supplemental material for Strategies for facilitating the delivery of cluster randomized trials in hospitals: A study informed by the CFIR-ERIC matching tool [file sj-pdf-1-ctj-10.1177_17407745211001504.pdf]

Appendix A:

Consolidated Framework for Implementation Research Constructs

Construct		Short Description
**I. INTERVENTION CHARACTERISTICS**		
A	Intervention Source	Perception of key stakeholders about whether the intervention is externally or internally developed.
B	Evidence Strength & Quality	Stakeholders' perceptions of the quality and validity of evidence supporting the belief that the intervention will have desired outcomes.
C	Relative Advantage	Stakeholders' perception of the advantage of implementing the intervention versus an alternative solution.
D	Adaptability	The degree to which an intervention can be adapted, tailored, refined, or reinvented to meet local needs.
E	Trialability	The ability to test the intervention on a small scale in the organization, and to be able to reverse course (undo implementation) if warranted.
F	Complexity	Perceived difficulty of implementation, reflected by duration, scope, radicalness, disruptiveness, centrality, and intricacy and number of steps required to implement.
G	Design Quality & Packaging	Perceived excellence in how the intervention is bundled, presented, and assembled.
H	Cost	Costs of the intervention and costs associated with implementing the intervention including investment, supply, and opportunity costs.
**II. OUTER SETTING**		
A	Patient Needs & Resources	The extent to which patient needs, as well as barriers and facilitators to meet those needs, are accurately known and prioritized by the organization.
B	Cosmopolitanism	The degree to which an organization is networked with other external organizations.
C	Peer Pressure	Mimetic or competitive pressure to implement an intervention; typically because most or other key peer or competing organizations have already implemented or are in a bid for a competitive edge.
D	External Policy & Incentives	A broad construct that includes external strategies to spread interventions, including policy and regulations (governmental or other central entity), external mandates, recommendations and guidelines, pay-for-performance, collaboratives, and public or benchmark reporting.
**III. INNER SETTING**		
A	Structural Characteristics	The social architecture, age, maturity, and size of an organization.

B	Networks & Communications	The nature and quality of webs of social networks and the nature and quality of formal and informal communications within an organization.
C	Culture	Norms, values, and basic assumptions of a given organization.
D	Implementation Climate	The absorptive capacity for change, shared receptivity of involved individuals to an intervention, and the extent to which use of that intervention will be rewarded, supported, and expected within their organization.
1	Tension for Change	The degree to which stakeholders perceive the current situation as intolerable or needing change.
2	Compatibility	The degree of tangible fit between meaning and values attached to the intervention by involved individuals, how those align with individuals' own norms, values, and perceived risks and needs, and how the intervention fits with existing workflows and systems.
3	Relative Priority	Individuals' shared perception of the importance of the implementation within the organization.
4	Organizational Incentives & Rewards	Extrinsic incentives such as goal-sharing awards, performance reviews, promotions, and raises in salary, and less tangible incentives such as increased stature or respect.
5	Goals and Feedback	The degree to which goals are clearly communicated, acted upon, and fed back to staff, and alignment of that feedback with goals.
6	Learning Climate	A climate in which: a) leaders express their own fallibility and need for team members' assistance and input; b) team members feel that they are essential, valued, and knowledgeable partners in the change process; c) individuals feel psychologically safe to try new methods; and d) there is sufficient time and space for reflective thinking and evaluation.
E	Readiness for Implementation	Tangible and immediate indicators of organizational commitment to its decision to implement an intervention.
1	Leadership Engagement	Commitment, involvement, and accountability of leaders and managers with the implementation.
2	Available Resources	The level of resources dedicated for implementation and on-going operations, including money, training, education, physical space, and time.
3	Access to Knowledge & Information	Ease of access to digestible information and knowledge about the intervention and how to incorporate it into work tasks.
**IV. CHARACTERISTICS OF INDIVIDUALS**		
A	Knowledge & Beliefs about the Intervention	Individuals' attitudes toward and value placed on the intervention as well as familiarity with facts, truths, and principles related to the intervention.
B	Self-efficacy	Individual belief in their own capabilities to execute courses of action to achieve implementation goals.

C	Individual Stage of Change	Characterization of the phase an individual is in, as he or she progresses toward skilled, enthusiastic, and sustained use of the intervention.
D	Individual Identification with Organization	A broad construct related to how individuals perceive the organization, and their relationship and degree of commitment with that organization.
E	Other Personal Attributes	A broad construct to include other personal traits such as tolerance of ambiguity, intellectual ability, motivation, values, competence, capacity, and learning style.
**V. PROCESS**		
A	Planning	The degree to which a scheme or method of behavior and tasks for implementing an intervention are developed in advance, and the quality of those schemes or methods.
B	Engaging	Attracting and involving appropriate individuals in the implementation and use of the intervention through a combined strategy of social marketing, education, role modeling, training, and other similar activities.
1	Opinion Leaders	Individuals in an organization who have formal or informal influence on the attitudes and beliefs of their colleagues with respect to implementing the intervention.
2	Formally Appointed Internal Implementation Leaders	Individuals from within the organization who have been formally appointed with responsibility for implementing an intervention as coordinator, project manager, team leader, or other similar role.
3	Champions	"Individuals who dedicate themselves to supporting, marketing, and 'driving through' an [implementation]" [101] (p. 182), overcoming indifference or resistance that the intervention may provoke in an organization.
4	External Change Agents	Individuals who are affiliated with an outside entity who formally influence or facilitate intervention decisions in a desirable direction.
C	Executing	Carrying out or accomplishing the implementation according to plan.
D	Reflecting & Evaluating	Quantitative and qualitative feedback about the progress and quality of implementation accompanied with regular personal and team debriefing about progress and experience.

Appendix B:

The Expert Recommendations for Implementing Change compilation

**Strategy**	**Definitions**
Access new funding	Access new or existing money to facilitate the implementation
Alter incentive/allowance structures	Work to incentivize the adoption and implementation of the clinical innovation
Alter patient/consumer fees	Create fee structures where patients/consumers pay less for preferred treatments (the clinical innovation) and more for less-preferred treatments
Assess for readiness and identify barriers and facilitators	Assess various aspects of an organization to determine its degree of readiness to implement, barriers that may impede implementation, and strengths that can be used in the implementation effort
Audit and provide feedback	Collect and summarize clinical performance data over a specified time period and give it to clinicians and administrators to monitor, evaluate, and modify provider behavior
Build a coalition	Recruit and cultivate relationships with partners in the implementation effort
Capture and share local knowledge	Capture local knowledge from implementation sites on how implementers and clinicians made something work in their setting and then share it with other sites
Centralize technical assistance	Develop and use a centralized system to deliver technical assistance focused on implementation issues
Change accreditation or membership requirements	Strive to alter accreditation standards so that they require or encourage use of the clinical innovation. Work to alter membership organization requirements so that those who want to affiliate with the organization are encouraged or required to use the clinical innovation
Change liability laws	Participate in liability reform efforts that make clinicians more willing to deliver the clinical innovation
Change physical structure and equipment	Evaluate current configurations and adapt, as needed, the physical structure and/or equipment (*e.g.*, changing the layout of a room, adding equipment) to best accommodate the targeted innovation
Change record systems	Change records systems to allow better assessment of implementation or clinical outcomes
Change service sites	Change the location of clinical service sites to increase access
Conduct cyclical small tests of change	Implement changes in a cyclical fashion using small tests of change before taking changes system-wide. Tests of change benefit from systematic measurement, and results of the tests of change are studied for insights on how to do better. This process continues serially over time, and refinement is added with each cycle
Conduct educational meetings	Hold meetings targeted toward different stakeholder groups (*e.g.*, providers, administrators, other organizational stakeholders, and community, patient/consumer, and family stakeholders) to teach them about the clinical innovation

Conduct educational outreach visits	Have a trained person meet with providers in their practice settings to educate providers about the clinical innovation with the intent of changing the provider's practice
Conduct local consensus discussions	Include local providers and other stakeholders in discussions that address whether the chosen problem is important and whether the clinical innovation to address it is appropriate
Conduct local needs assessment	Collect and analyze data related to the need for the innovation
Conduct ongoing training	Plan for and conduct training in the clinical innovation in an ongoing way
Create a learning collaborative	Facilitate the formation of groups of providers or provider organizations and foster a collaborative learning environment to improve implementation of the clinical innovation
Create new clinical teams	Change who serves on the clinical team, adding different disciplines and different skills to make it more likely that the clinical innovation is delivered (or is more successfully delivered)
Create or change credentialing and/or licensure standards	Create an organization that certifies clinicians in the innovation or encourage an existing organization to do so. Change governmental professional certification or licensure requirements to include delivering the innovation. Work to alter continuing education requirements to shape professional practice toward the innovation
Develop a formal implementation blueprint	Develop a formal implementation blueprint that includes all goals and strategies. The blueprint should include the following: 1) aim/purpose of the implementation; 2) scope of the change (*e.g.*, what organizational units are affected); 3) timeframe and milestones; and 4) appropriate performance/progress measures. Use and update this plan to guide the implementation effort over time
Develop academic partnerships	Partner with a university or academic unit for the purposes of shared training and bringing research skills to an implementation project
Develop an implementation glossary	Develop and distribute a list of terms describing the innovation, implementation, and stakeholders in the organizational change
Develop and implement tools for quality monitoring	Develop, test, and introduce into quality-monitoring systems the right input—the appropriate language, protocols, algorithms, standards, and measures (of processes, patient/consumer outcomes, and implementation outcomes) that are often specific to the innovation being implemented
Develop and organize quality monitoring systems	Develop and organize systems and procedures that monitor clinical processes and/or outcomes for the purpose of quality assurance and improvement
Develop disincentives	Provide financial disincentives for failure to implement or use the clinical innovations
Develop educational materials	Develop and format manuals, toolkits, and other supporting materials in ways that make it easier for stakeholders to learn about the innovation and for clinicians to learn how to deliver the clinical innovation
Develop resource sharing agreements	Develop partnerships with organizations that have resources needed to implement the innovation

Distribute educational materials	Distribute educational materials (including guidelines, manuals, and toolkits) in person, by mail, and/or electronically
Facilitate relay of clinical data to providers	Provide as close to real-time data as possible about key measures of process/outcomes using integrated modes/channels of communication in a way that promotes use of the targeted innovation
Facilitation	A process of interactive problem solving and support that occurs in a context of a recognized need for improvement and a supportive interpersonal relationship
Fund and contract for the clinical innovation	Governments and other payers of services issue requests for proposals to deliver the innovation, use contracting processes to motivate providers to deliver the clinical innovation, and develop new funding formulas that make it more likely that providers will deliver the innovation
Identify and prepare champions	Identify and prepare individuals who dedicate themselves to supporting, marketing, and driving through an implementation, overcoming indifference or resistance that the intervention may provoke in an organization
Identify early adopters	Identify early adopters at the local site to learn from their experiences with the practice innovation
Increase demand	Attempt to influence the market for the clinical innovation to increase competition intensity and to increase the maturity of the market for the clinical innovation
Inform local opinion leaders	Inform providers identified by colleagues as opinion leaders or "educationally influential" about the clinical innovation in the hopes that they will influence colleagues to adopt it
Develop academic partnerships	Partner with a university or academic unit for the purposes of shared training and bringing research skills to an implementation project
Develop an implementation glossary	Develop and distribute a list of terms describing the innovation, implementation, and stakeholders in the organizational change
Intervene with patients/consumers to enhance uptake and adherence	Develop strategies with patients to encourage and problem solve around adherence
Involve executive boards	Involve existing governing structures (*e.g.*, boards of directors, medical staff boards of governance) in the implementation effort, including the review of data on implementation processes
Involve patients/consumers and family members	Engage or include patients/consumers and families in the implementation effort
Make billing easier	Make it easier to bill for the clinical innovation
Make training dynamic	Vary the information delivery methods to cater to different learning styles and work contexts, and shape the training in the innovation to be interactive
Mandate change	Have leadership declare the priority of the innovation and their determination to have it implemented
Model and simulate change	Model or simulate the change that will be implemented prior to implementation

Obtain and use patients/consumers and family feedback	Develop strategies to increase patient/consumer and family feedback on the implementation effort
Obtain formal commitments	Obtain written commitments from key partners that state what they will do to implement the innovation
Organize clinician implementation team meetings	Develop and support teams of clinicians who are implementing the innovation and give them protected time to reflect on the implementation effort, share lessons learned, and support one another's learning
Place innovation on fee for service lists/formularies	Work to place the clinical innovation on lists of actions for which providers can be reimbursed (*e.g.*, a drug is placed on a formulary, a procedure is now reimbursable)
Prepare patients/consumers to be active participants	Prepare patients/consumers to be active in their care, to ask questions, and specifically to inquire about care guidelines, the evidence behind clinical decisions, or about available evidence-supported treatments
Promote adaptability	Identify the ways a clinical innovation can be tailored to meet local needs and clarify which elements of the innovation must be maintained to preserve fidelity
Promote network weaving	Identify and build on existing high-quality working relationships and networks within and outside the organization, organizational units, teams, etc. to promote information sharing, collaborative problem-solving, and a shared vision/goal related to implementing the innovation
Provide clinical supervision	Provide clinicians with ongoing supervision focusing on the innovation. Provide training for clinical supervisors who will supervise clinicians who provide the innovation
Provide local technical assistance	Develop and use a system to deliver technical assistance focused on implementation issues using local personnel
Provide ongoing consultation	Provide ongoing consultation with one or more experts in the strategies used to support implementing the innovation
Purposely re-examine the implementation	Monitor progress and adjust clinical practices and implementation strategies to continuously improve the quality of care
Recruit, designate, and train for leadership	Recruit, designate, and train leaders for the change effort
Remind clinicians	Develop reminder systems designed to help clinicians to recall information and/or prompt them to use the clinical innovation
Revise professional roles	Shift and revise roles among professionals who provide care, and redesign job characteristics
Shadow other experts	Provide ways for key individuals to directly observe experienced people engage with or use the targeted practice change/innovation
Stage implementation scale up	Phase implementation efforts by starting with small pilots or demonstration projects and gradually move to a system wide rollout
Start a dissemination organization	Identify or start a separate organization that is responsible for disseminating the clinical innovation. It could be a for-profit or non-profit organization
Tailor strategies	Tailor the implementation strategies to address barriers and leverage facilitators that were identified through earlier data collection

Use advisory boards and workgroups	Create and engage a formal group of multiple kinds of stakeholders to provide input and advice on implementation efforts and to elicit recommendations for improvements
Use an implementation advisor	Seek guidance from experts in implementation
Use capitated payments	Pay providers or care systems a set amount per patient/consumer for delivering clinical care
Use data experts	Involve, hire, and/or consult experts to inform management on the use of data generated by implementation efforts
Use data warehousing techniques	Integrate clinical records across facilities and organizations to facilitate implementation across systems
Use mass media	Use media to reach large numbers of people to spread the word about the clinical innovation
Use other payment schemes	Introduce payment approaches (in a catch-all category)
Use train-the-trainer strategies	Train designated clinicians or organizations to train others in the clinical innovation
Visit other sites	Visit sites where a similar implementation effort has been considered successful
Work with educational institutions	Encourage educational institutions to train clinicians in the innovation
